# Helicase-like transcription factor: a new marker of well-differentiated thyroid cancers

**DOI:** 10.1186/1471-2407-14-492

**Published:** 2014-07-08

**Authors:** Vanessa Arcolia, Paula Paci, Ludovic Dhont, Gilbert Chantrain, Nicolas Sirtaine, Christine Decaestecker, Myriam Remmelink, Alexandra Belayew, Sven Saussez

**Affiliations:** 1 Laboratory of Anatomy and Cell Biology, Faculty of Medicine and Pharmacy, Research Institute for Health Sciences and Technology, University of Mons, 7000 Mons, Belgium; 2 Laboratory of Molecular Biology, Faculty of Medicine and Pharmacy, Research Institute for Health Sciences and Technology, University of Mons, 7000 Mons, Belgium; 3 Department of Oto-Rhino-Laryngology, CHU Saint-Pierre, Free University of Brussels, Brussels, Belgium; 4 Department of Pathology, Institute Bordet, Free University of Brussels, Brussels, Belgium; 5 Laboratory of Image, Signal Processing and Acoustics (LISA), Ecole Polytechnique de Bruxelles, Free University of Brussels, Brussels, Belgium; 6 Department of Pathology, Hospital Erasme, Free University of Brussels, Brussels, Belgium; 7 Present address: Laboratory of Neurosciences, Faculty of Medicine and Pharmacy, Research Institute for Health Sciences and Technology, University of Mons, 7000 Mons, Belgium

**Keywords:** Biomarker, HLTF, Diagnosis, Tumor suppressor, Thyroid lesions, Cancer

## Abstract

**Background:**

The preoperative characterization of thyroid nodules is a challenge for the clinicians. Fine-needle aspiration (FNA) is the commonly used pre-operative technique for diagnosis of malignant thyroid tumor. However, many benign lesions, with indeterminate diagnosis following FNA, are referred to surgery. There is an urgent need to identify biomarkers that could be used with the FNA to distinguish benign thyroid nodules from malignant tumors. The purpose of the study is to examine the level of expression of the helicase-like transcription factor (HLTF) in relation to neoplastic progression of thyroid carcinomas.

**Methods:**

The presence of HLTF was investigated using quantitative and semi-quantitative immunohistochemistry in a series of 149 thyroid lesion specimens. Our first clinical series was composed of 80 patients, including 20 patients presenting thyroid adenoma, 40 patients presenting thyroid papillary carcinoma, 12 patients presenting thyroid follicular carcinoma and 8 patients presenting anaplastic carcinoma. These specimens were assessed quantitatively using computer assisted microscopy. Our initial results were validated on a second clinical series composed of 40 benign thyroid lesions and 29 malignant thyroid lesions using a semi-quantitative approach. Finally, the HLTF protein expression was investigated by Western blotting in four thyroid cancer cell lines.

**Results:**

The decrease of HLTF staining was statistically significant during thyroid tumor progression in terms of both the percentage of mean optical density (MOD), which corresponds to the mean staining intensity (Kruskall-Wallis: p < 0.0005), and the labelling index (LI), which corresponds to the percentage of immunopositive cells (Kruskall-Wallis: p < 10^−6^). Adenomas presented very pronounced nuclear HLTF immunostaining, whereas papillary carcinomas exhibited HLTF only in the cytoplasm. The number of HLTF positive nuclei was clearly higher in the adenomas group (30%) than in the papillary carcinomas group (5%).

The 115-kDa full size HLTF protein was immunodetected in four studied thyroid cancer cell lines. Moreover, three truncated HLTF forms (95-kDa, 80-kDa and 70-kDa) were also found in these tumor cells.

**Conclusions:**

This study reveals an association between HLTF expression level and thyroid neoplastic progression. Nuclear HLTF immunostaining could be used with FNA in an attempt to better distinguish benign thyroid nodules from malignant tumors.

## Background

Thyroid cancer is the most common malignant endocrine tumor with an estimated annual incidence of 122,803 cases worldwide [1]. Well-differentiated thyroid carcinomas (papillary and follicular carcinomas) have an excellent prognosis, with 85 to 90% cure rates thanks to early detection and appropriate treatment. Until now, fine-needle aspiration (FNA) was the most commonly used pre-operative technique for diagnosis of a malignant thyroid tumor. However, using ultrasound-guided FNA, this technique showed inconclusive biopsy results in 10-20% of all cases, even with the best hands of radiologist and/or pathologist. Thus, there is an urgent need to identify new biomarkers that could be used in conjunction with the FNA to distinguish benign thyroid nodules from malignant tumors. Such biomarkers may provide crucial knowledge about the biology of well-differentiated thyroid cancers and, most likely, new directions for targeted therapies.

Helicase-like Transcription Factor (HLTF) is a SWI/SNF (mating-type switching/sucrose non-fermenting) protein that presents 7 DNA helicase domains that use the energy of ATP hydrolysis to remodel chromatin in a variety of cellular processes; it was initially characterized for its DNA binding and transcriptional activity [2,3]. HLTF was later shown to act as an E3 ubiquitin ligase implicated in post-replication DNA repair by polyubiquitination of proliferating cell nuclear antigen (PCNA) and to be recruited to chromatin by the BRCA1 tumor suppressor together with translesion synthesis polymerases [4-7]. HLTF, as well as its yeast Rad5 ortholog, promote the repair of gaps formed at stalled replication forks on damaged DNA [8]. The inactivation of HLTF induces an elevated level of chromosome breaks and fusions in mouse embryonic fibroblasts after treatment with alkylating agents [5] and increases the sensitivity of cells submitted to DNA damaging agents. In agreement with a post-replicative function, HLTF inactivation in a transgenic mouse disrupts cell cycle progression at the G2-M transition both in heart and brain cells [9,10].

Several studies have demonstrated that the *HLTF* promoter is hypermethylated in human colorectal [11-19], gastric [13,20,21], esophageal [13,22] and uterine cancers [23], suggesting that *HLTF* silencing may play a crucial role in cancer. Moreover, H. Ding’s group confirmed a tumor suppressor function by mouse transgenesis: HLTF deficiency in *Apc*^*−/+*^ mice induced the transition from colon adenoma to carcinoma with high chromosomal instability [24]. In a different model, HLTF induction was detected very early in small pre-neoplastic buds of hamster kidney tumors induced by diethylstilbestrol [25]. Recently, we studied the immunohistochemical expression of HLTF in relation to head and neck squamous cell carcinoma (HNSCC) tumor progression. We showed that HLTF expression increases significantly when comparing carcinomas to normal epithelia or dysplasias [26]. Moreover, high levels of HLTF expression were found to be associated with rapid recurrence rates in a series of 100 hypopharyngeal squamous cell carcinomas (SCC) [26].

We characterized the HLTF mRNAs from HeLa cells by RT-PCR and identified 6 protein forms. One of these forms, the 95-kDa HLTF, was detected along with the 115-kDa full-length protein in both head and neck SCC biopsies. The 95-kDa variant did not contain the 3 carboxyl-terminal helicase domains that are involved in DNA repair [26]. Similarly, the immunoexpression of the 115-kDa full-size HLTF protein increased during carcinogenesis of the uterine cervix but was replaced by truncated forms (95 or 83-kDa) in squamous cell carcinoma [27]. These observations suggested that HLTF expression could also be altered in thyroid carcinogenesis.

## Methods

**Figure 1:**
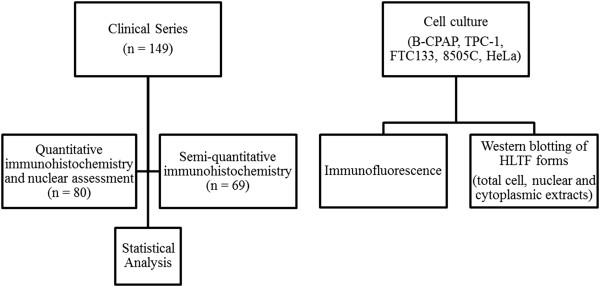
**Experimental flow diagram.** The immunohisto/cytochemical HLTF expression was studied in a clinical series of 149 patients (with benign and malignant thyroid lesions) and on thyroid cancer cell lines.

The first part of the study was conducted using thyroid tumor surgical specimens. Immunohistochemical HLTF expression was studied in a clinical series of 149 patients with benign and malignant thyroid lesions. The pathological area was analyzed quantitatively by computer assisted morphometry in 80 cases (in addition to a nuclear assessment) and semi-quantitatively in 69 additional cases. These data were submitted to statistical analysis (Figure 1).The second part of the study was performed using thyroid cancer cell lines (B-CPAP, TPC-1, FTC133, 8505C and HeLa). Immunocytochemical HLTF expression was examined by immunofluorescence and Western blotting (Figure 1).

### Patient’ characteristics

The immunohistochemical HLTF expression was first studied in a clinical series of 80 patients (23 males and 57 females), including 20 patients with thyroid adenoma, 40 patients presenting with thyroid papillary carcinoma, 12 patients presenting with thyroid follicular carcinoma and 8 patients presenting with anaplastic carcinoma. This series was selected by a pathologist (Prof. M. Remmelink, Erasme hospital, Department of Pathology, ULB, Belgium), and after her delineation, the pathological area was analyzed quantitatively by computer-assisted morphometry.

For the second part, we extended our analysis to a series of 40 patients (9 males and 60 females) with benign thyroid lesions, including 10 colloid nodules, 16 follicular adenomas, 7 Hashimoto’s thyroiditis, and 7 Grave’s disease, and to a series of 29 patients with malignant thyroid lesions including 17 papillary carcinomas and 12 follicular variant of papillary carcinomas. These series were selected by the same pathologist (Prof. M. Remmelink, Erasme hospital, Department of Pathology, ULB, Belgium), and after selection, the pathological area was analyzed semi-quantitatively.

### Ethics Statement

These clinical studies were approved by the Faculty of Medicine and Pharmacy (University of Mons, Belgium) ethics committee (OM 004). A written informed consent was obtained from all patients.

### Anti-HLTF antibodies

Two human HLTF protein forms are expressed from the same open reading frame and differ only in the translation start site (Met1 or Met123) [2]. The ART2 rabbit anti-serum was raised against a peptide in the HLTF amino-terminal sequence specific of the full length Met1 form, as previously described in Debauve *et al*. [25].

The anti-COOH rabbit serum (Eurogentec) is specific to the wild type HLTF proteins (Met1 and Met123) and was raised against a peptide (CFGTKKPNADEMKQAX) that is missing in the HLTF truncated variants.

The anti-HLTF rabbit serum (HPA015284, Sigma) detects all HLTF protein forms and was raised against a peptide extending from residues 164 to 300 (LKKHG…MGLGK).

### Cell Culture

Four cell lines derived from human thyroid cancers were studied: B-CPAP and TPC-1 derived from papillary carcinoma (Dr. C.Paulin, Laboratory of Anatomy and Cytology-Pathology, University of Lyon, Lyon, France and Prof. C.Maenhout, IRIBHM ULB, Brussels, Belgium, respectively), FTC-133 derived from follicular carcinoma (Dr. Köhrle, Institute of Experimental Endocrinology of the Charité, Humboldt University, Berlin, Germany) and 8505C derived from anaplastic carcinoma (Dr. Akiyama, Radiation Effects Research Foundation, Hiroshima). The HeLa cell line was used as the positive control. HeLa cells are derived from cervical carcinoma and are routinely cultivated in the laboratory of Molecular Biology (UMons). The cell lines HeLa, FTC-133, TPC-1, B-CPAP and 8505C were grow in Dulbecco’s Modified Essential Medium–F12 (DMEM-F12, Lonza) and RPMI 1640 (Lonza) with 10% fetal bovine serum (FBS, PAA Laboratories) and 1% antibiotics and antimycotics (PAA Laboratories). The cells were incubated at 37°C with 5% CO_2_. The culture medium was changed once every 3 days. For routine subculture and cell plating in preparation for immunofluorescence studies, cells were detached by incubation with Accutase (PAA Laboratories), resuspended and counted using a hemocytometer.

### Immunofluorescence Staining of Cultured Cells

Six-well culture plates containing sterile round glass coverslips were seeded at a density of 5x10^5^ cells/well and grown for 24 h. Cell cultures were rinsed with phosphate-buffered saline (PBS: 0.04 M Na_2_HPO_4_, 0.01 M KH_2_PO_4_ and 0.12 M NaCl, pH 7.4) and fixed in 4% paraformaldehyde for 10 min at 4°C and 5 min at room temperature (RT). The fixed cells were rinsed in PBS and blocked by incubation with bovine serum albumin (PBS plus 0.2% Triton X-100 and 5% BSA) for 30 min at RT. The fixed cells were incubated in the ART2 primary antibody diluted 1/100 in the blocking buffer for 1 h at RT, rinsed in PBS and incubated with Alexa Fluor 555 goat anti-rabbit IgG (Invitrogen) for 30 min. The immunostained sections were rinsed in PBS, mounted in Vectashield (Vector Laboratories), examined under a LeitzOrthoplan fluorescence microscope (Ploem system) and representative fields were recorded with a digital camera (Leica DC 300 F). Controls for the immunostaining specificity included the omission of the primary antibody or the substitution of non-immune sera for the primary antibodies. The specificity of anti-HLTF immunostaining was also examined with primary antibodies previously incubated with the synthetic peptide used as an antigen. In each case, these controls were negative (data not shown).

### Immunohistochemistry of Benign and Malignant Thyroid Tumors

All tumor samples were fixed for 24 h in 10% buffered formaldehyde, dehydrated and then embedded in paraffin. Immunochemistry was performed on 5µm-thick sections that were mounted on silane-coated glass sides. Before initiating the immunochemistry, tissue sections were briefly exposed to microwave pre-treatment in a 0.01 M citrate buffer (pH 6.2) for 2 × 5 min at 900 W. The sections were then incubated in a 0.06% hydrogen peroxide solution for 4 min to block endogenous peroxidase activity, rinsed in PBS and successively exposed for 5 min to solutions containing avidin (0.1 mg/ml PBS) and biotin (0.1 mg/ml PBS) to avoid false-positive staining reactions from endogenous biotin. After a washing step with PBS, the sections were incubated for 15 min with a solution of 0.5% casein in PBS and sequentially exposed at RT to solutions of i) specific primary anti-HLTF antibody (1/50); ii) corresponding biotinylated secondary antibody (polyclonal goat anti-rabbit IgG, 1/50, Vector Laboratories, Burlingame, CA); and iii) avidin- biotin-peroxidase complex (ABC Kit, Vector Laboratories, Burlingame, CA). The incubation steps were alternated with washing steps to remove unbound proteins. Antigen-dependent presence of the peroxidase complex in the sections was visualized by incubation with chromogenic substrates containing diaminobenzidine and H_2_O_2_. After rinsing, the sections were counterstained with Luxol fast blue and mounted with a synthetic medium. To exclude antigen-independent staining, the incubation step with primary antibodies was omitted from the protocol in negative controls. In all cases, these controls were negative (data not shown).

### Immunohistochemical Quantitative Analysis using a Computer-assisted microscopy

After the immunohistochemical steps, the levels of HLTF protein expression were quantitatively determined in our initial clinical series using a computer-assisted KS 400 imaging system (Carl Zeiss Vision, Hallbergmoos, Germany) connected to a Zeiss Axioplan microscope. For each section, 10 fields were analyzed at a 20X magnification and a picture was recorded for each of them by the ProgResCapturePro 2.1 program. The morphometric analysis was focused on selected pathological areas where two variables were measured: the labelling index (LI) and the mean optical density (MOD). The median was calculated for each section and for each tumor type.

### Nuclear assessment by Optical Microscopy

For each specimen, 10 fields were analyzed at a 40X magnification to count the immunopositive nuclei and also the total number of nuclei in a gate of 0.084 mm^2^. The mean of positive nuclei was assessed for each specimen.

### Immunohistochemical Semi-quantitative Analysis

After the immunohistochemical steps, the levels of HLTF expression were semi-quantitatively determined on the second clinical series in collaboration with a pathologist (M.R) using an optical microscope (Leitz). The mean intensity (MI) was defined as follows: 0 (negative), 1 (low), 2 (moderate), and 3 (strong). The percentages of immunopositive tissue areas (Labelling index, LI) were categorized as follows: 0 (0% positive cells), 1 (1-25%), 2 (26-75%), and 3 (76-100%).

### Western blotting

Total cell, nuclear and cytoplasmic extracts were used for all cell lines. To obtain total cell extracts, cells were recovered on ice with a scraper in 1 ml of PBS. The cell suspension was centrifuged at 4000 rpm for 5 min at 4°C. We resuspended the pellet in 100µl of lysis buffer (BugBuster Protein Extraction Reagent, Novagen) for protein extraction. Nuclear and cytoplasmic extractions were performed using the NE-PER Nuclear and Cytoplasmic Extraction kit (Thermo Fisher Scientific) according to the manufacturer’s instructions.

Cells were homogenized at 4°C in hypertonic buffer (Tris 50 mM pH 7, NaCl 500 mM and NP40 0.1%) with added anti-proteases (Protease Inhibitor Cocktail, Sigma) and 1 mM DTT. Protein concentrations were measured using the Bio-Rad protein assay (BioRad Laboratories). 40µg (for nuclear and cytoplasmic extracts) or 60µg (for total cell extracts) of protein were diluted in 5µl 4X LDS sample buffer (NuPAGE LDS sample buffer 4X, Invitrogen), 1µl 20X reducing reagent (Fermentas) and up to 20µl water, heated to 95°C for 5 min and separated by 4-12% PAGE-SDS (NuPAGE 4%-12% Bis-Tris Gel, Novex). After electrophoresis, the proteins were electro-transferred from the gel onto a nitrocellulose membrane (Hybond ECL, Amersham Pharmacia Biotech) using an electrophoretic transfer cell (Bio-Rad) at 260 mA for 1 h 45 min at 4°C. The transfer efficiency was controlled by Ponceau red staining (Ponceau S Solution, Sigma). Non-specific protein binding sites on the membrane were blocked by incubation with PBS 5% non-fat milk (Bio-Rad) for 1 h at RT. The membrane was incubated at 4°C overnight in the anti-HLTF primary antibody (Sigma) diluted 1/500 (for nuclear and cytoplasmic extracts) or 1/1000 (for total cell extracts) in the blocking buffer. After incubation, the membrane was washed three times with PBS and incubated for 1 h at RT with HRP-conjugated goat anti-rabbit IgG antibodies (Amersham Pharmacia Biotech) diluted 1/5000 in 0.5% Albumin bovine fraction V (BSA, MP Biomedicals). After treatment with Super Signal West Femto Chemiluminescent Substrate (Thermo Scientific), the membrane was exposed for 15 min on a photosensitive film (Hyperfilm ECL, Amersham Pharmacia Biotech). Molecular weight markers were analyzed in parallel for internal calibration (PAGE Ruler Prestained Proteins, Fermentas).

### Statistical Analysis

Independent groups of quantitative and semi-quantitative data were compared using the nonparametric Kruskall-Wallis test (for comparison of more than two groups). In the case of more than two groups, post-hoc tests (Dunn procedure) were used to compare pairs of groups (to avoid multiple comparison effects).

## Results

### HLTF immunostaining decreases with tumor progression and shifts from the nucleus to the cytoplasm

**Figure 2:**
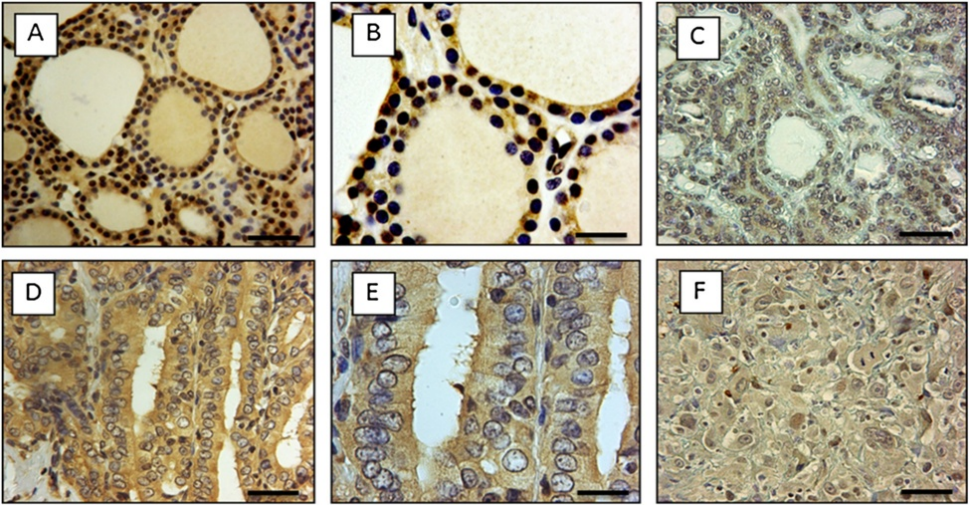
**Immunohistochemical staining of HLTF (ART2 rabbit serum) in benign and malignant thyroid tumors. A**-**B**: adenoma, **C**: follicular carcinoma, **D**-**E**: papillary carcinoma and **F**: anaplastic carcinoma. The scale bar is 25µm **(B and E)** and 50µm **(A, C, D and F)**.

The first aim of our study was to quantitatively investigate HLTF expression in a series of 80 thyroid tumors. Moreover, the HLTF sub-location (nuclear staining) was also assessed by cell counting. In adenomas, the HLTF staining was moderate in the cytoplasm, but a pronounced nuclear staining was clearly observed (Figure 2A, B). In contrast, in papillary carcinomas, the HLTF staining was limited to the cytoplasm (Figure 2D, E), whereas follicular and anaplastic carcinomas exhibited weak cytoplasmic and nuclear immunostaining (Figure 2C, F, respectively).

**Figure 3:**
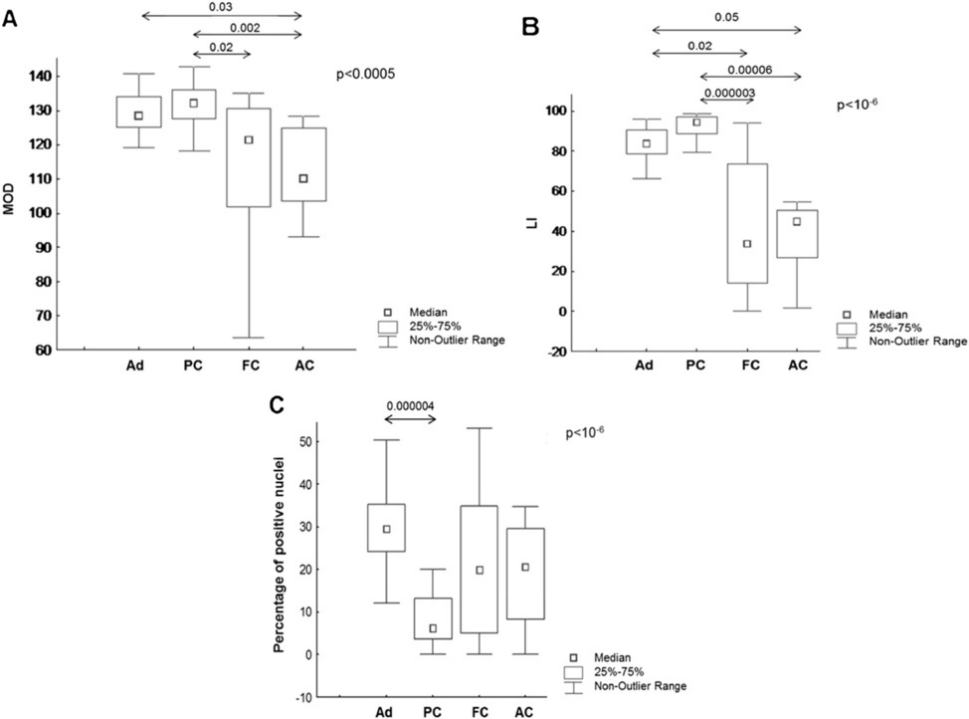
**Statistical analysis of HLTF staining in a series of 4 groups of thyroid tumors.** Ad: thyroid adenomas, PC: papillary carcinomas, FC: follicular carcinomas and AC: anaplastic carcinomas. The immunohistochemical expression was assessed quantitatively using the MOD **(A)**, LI **(B)** and the percentage of positive nuclei **(C)**. The threshold of significance associated with the global multigroup comparison (Kruskal-Wallis test) is located in the higher right corner. Horizontal arrows represent the post-hoc comparison for the pairs of groups with corresponding p-values.

To clearly present our data, we first compared all groups (Adenomas: Ad; Papillary Carcinomas: PC; Follicular Carcinomas: FC; and Anaplastic carcinomas: AC) using a nonparametric Kruskall-Wallis test (Figure 3). The decrease in HLTF staining was statistically significant during thyroid tumor progression in terms of both MOD, which corresponds to the mean staining intensity (Kruskall-Wallis: p < 0.0005, Figure 3A), and the LI, which corresponds to the percentage of immunopositive cells (Kruskall-Wallis: p < 10^−6^, Figure 3B). Using a post-hoc comparison for pairs of groups, we observed that HLTF expression decreased when comparing adenomas to anaplastic carcinomas (MOD: p = 0.03, Figure 3A; LI: p = 0.05, Figure 3B), adenomas to follicular carcinomas (LI: p = 0.02, Figure 3B), papillary carcinomas to follicular carcinomas (MOD: p = 0.02, Figure 2A; LI: p = 0.000003, Figure 2B) and papillary carcinomas to anaplastic carcinomas (MOD: p = 0.002, Figure 3A; LI: p = 0.00006, Figure 3B).

In addition, the percentage of HLTF positive nuclei differed significantly among the 4 tumor groups (Kruskal-Wallis p < 10^−6^, Figure 3C). More precisely, this difference is significant when comparing adenomas to papillary carcinomas (p = 0.000004, post-hoc test, Figure 3C). Indeed, the median value of the number of HLTF positive nuclei in the adenoma group (30%) is clearly higher than that in the papillary carcinoma group (5%).

### HLTF immunostaining is detected in benign thyroid lesions

**Figure 4:**
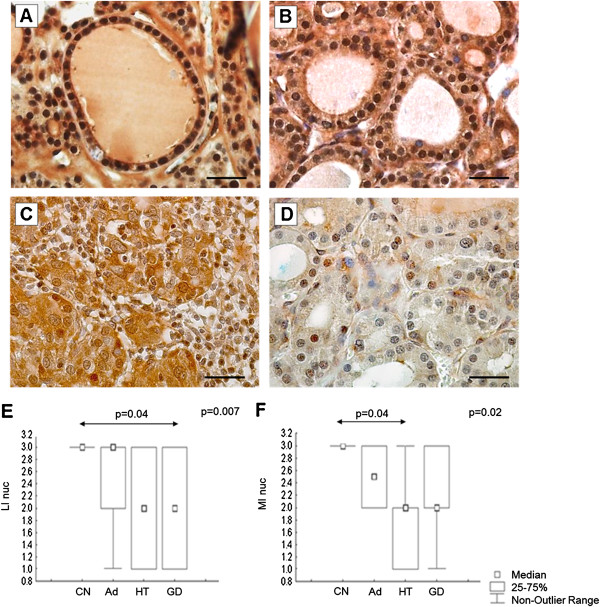
**Immunohistochemical staining of HLTF in benign thyroid lesions and statistical analysis of HLTF staining in a series of 4 groups of benign thyroid lesions. (A-D) A**: colloid nodule, **B**: adenoma, **C**: Hashimoto’s Thyroiditis and D: Graves’ Disease. The scale bar is 50µm. **(E-F)** CN: colloid nodules, Ad: follicular adenomas HT: Hashimoto’s Thyroiditis and GD: Graves’ Diseases. The immunohistochemical expression was semi-quantitatively assessed in the nuclear compartment using **(E)** the LI (LI nuc) and **(F)** the MI (MI nuc). The threshold of significance associated with the global multigroup comparison (Kruskal-Wallis test) is located in the higher right corner. Horizontal arrows represent the post-hoc comparison for the pairs of groups, with corresponding p-values.

Figure 4A-F illustrates the HLTF immunohistochemical expression in a series of 40 benign thyroid lesions including 10 with colloid nodules (CN), 16 with adenomas (Ad), 7 with Hashimoto’s thyroiditis (HT) and 7 with Grave’s disease (GD). In colloid nodules and adenomas, HLTF is localized in both the nucleus and the cytoplasm of follicular cells, but with a significantly higher staining intensity at the nuclear level (Figure 4A, B). In Hashimoto’s thyroiditis (HT) and Grave’s disease (GD), a decrease in nuclear staining was observed (Figure 4C, D, respectively). All of the specimens were assessed using a semi-quantitative analysis with one pathologist (MR) because the quantitative assessment could not be proposed in a routine examination. We compared all groups (CN, Ad, HT, GD) using a nonparametric Kruskall-Wallis test and obtained a significant difference in terms of both the percentage of the mean immunopositive nuclei (LI nuc: p = 0.007; Kruskal-Wallis) (Figure 4E) and the mean staining intensity of the immunopositive nuclei (MI nuc: p = 0.02; Kruskal-Wallis) (Figure 4F). These two parameters were decreased in Hashimoto’s thyroiditis (HT) and Grave’s disease (GD) when compared to colloid nodules (CN) and adenomas (Ad). Then, using a post-hoc comparisons for pairs of groups, we determined that the percentage of immunopositive epithelial cells was higher in colloid nodules than in Grave’s Disease (LI nuc: p = 0.04, post- hoc test) (Figure 4A, D, E). We also observed that the staining intensity of the immunopositive epithelial cells of colloid nodules was stronger than that in Hashimoto’s thyroiditis, where the nuclear staining was moderate (MI nuc: p = 0.04, post-hoc test) (Figure 4A, C, F). These results show that the HLTF expression level decreases in thyroid autoimmune diseases, which can develop into thyroid carcinomas, suggesting a potential role in early diagnosis.

### HLTF nuclear immunostaining profile during thyroid tumor progression

**Figure 5:**
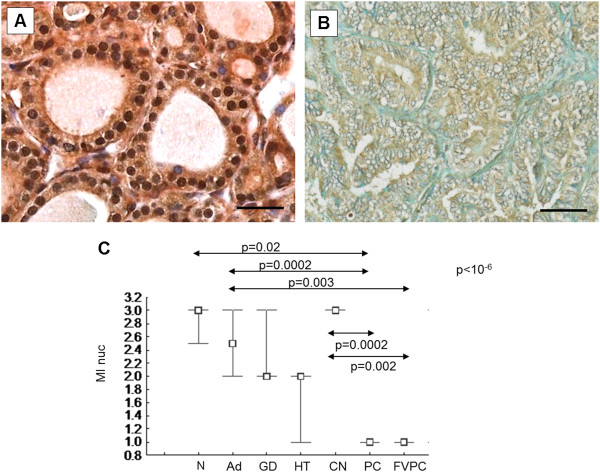
**Immunohistochemical staining of HLTF in benign and malignant thyroid tumors and statistical analysis of HLTF staining in benign and malignant thyroid lesions. (A-B) A**: follicular adenoma and **B**: papillary carcinoma. The scale bar is 50µm. **(C)** N: normal tissues, Ad: adenomas, HT: Hashimoto’s Thyroiditis, GD: Graves’ Diseases, CN: colloid nodules, PC: papillary carcinomas and FVPC: follicular variant of papillary carcinomas. The immunohistochemical expression was semi-quantitatively assessed in the nuclear compartment using the MI (MI nuc). The threshold of significance associated with the global multigroup comparison (Kruskal-Wallis test) is located in the higher right corner. Horizontal arrows represent the post-hoc comparison for the pairs of groups, with corresponding p-values.

The immunohistochemical expression of HLTF was investigated in a series of normal thyroid tissue as well as in benign and malignant lesions, more specifically, 4 normal thyroid tissue (N), 10 colloid nodules (CN), 16 adenomas (Ad), 7 Hashimoto’s thyroiditis (HT), 7 Grave’s disease (GD), 17 papillary carcinomas (PC) and 12 follicular variant of papillary carcinomas (FVPC). In adenomas, HLTF was localized in both the nucleus and the cytoplasm of cells but with significantly higher staining intensity at the nuclear level (Figure 5A). In papillary carcinomas, the HLTF protein was detected in both compartments but with a more pronounced staining at the nuclear periphery (Figure 5B). The intensity of nuclear staining by our anti-HLTF antibody was compared between these 7 groups for which a significant difference was detected (MI nuc: p < 10^−6^, Kruskal-Wallis) (Figure 5C). Post-hoc comparisons detected a significant difference between papillary carcinomas and normal tissue (MI nuc: p = 0.02; post-hoc test) and adenomas (MI nuc: p = 0.0002; post-hoc test) and colloid nodules (MI nuc: p = 0.0002; post-hoc test) (Figure 5C). We also observed a significant difference when we compared FVPC with adenomas (MI nuc: p = 0.003) or colloid nodules (MI nuc: p = 0.002) (Figure 5C). The intensity of the HLTF nuclear staining was strong in adenomas (MI nuc: median = 2.5) and decreased in papillary carcinomas (MI nuc: median = 1). These results corroborate the first observation in which HLTF expression decreased during thyroid tumor progression with a gradual shift of HLTF staining from the nucleus to the cytoplasm in malignant cells.

### Truncated HLTF protein forms are expressed in adenomas and papillary carcinomas

**Figure 6:**
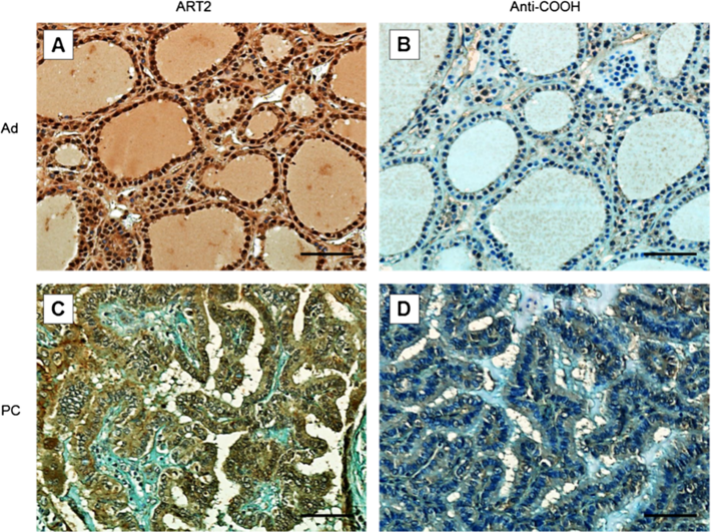
**Immunodetection of truncated HLTF forms lacking the carboxy-terminal domain.** Immunohistochemical detection of HLTF using rabbit antisera directed against the amino (ART2) or carboxy-terminal (anti-COOH) domains of the wild type protein on a biopsy of a follicular adenoma (Ad, **A**-**B**) or a papillary carcinoma (PC, **C**-**D**). The scale bar is 100µm.

HLTF immunohistochemical expression was investigated in biopsies of adenomas and papillary carcinomas using two different anti-HLTF antibodies. The ART2 serum targets the amino-terminal domain of full-length HLTF and two truncated HLTF forms (83- and 95- kDa) [26], whereas the anti-COOH serum targets the carboxy-terminal domain of wild-type HLTF that is missing in the truncated forms (see Methods). Using the ART2 antibody, HLTF was detected in both the nucleus and the cytoplasm of follicular cells in adenomas (Figure 6A). In papillary carcinomas, the staining was limited to the cytoplasm (Figure 6C). By contrast, with the anti-COOH antibody, HLTF staining was abolished both in adenomas and papillary carcinomas (Figure 6B, D), demonstrating the expression of truncated forms that lack DNA repair ability.

### HLTF staining pattern in thyroid cancer cell lines

**Figure 7:**
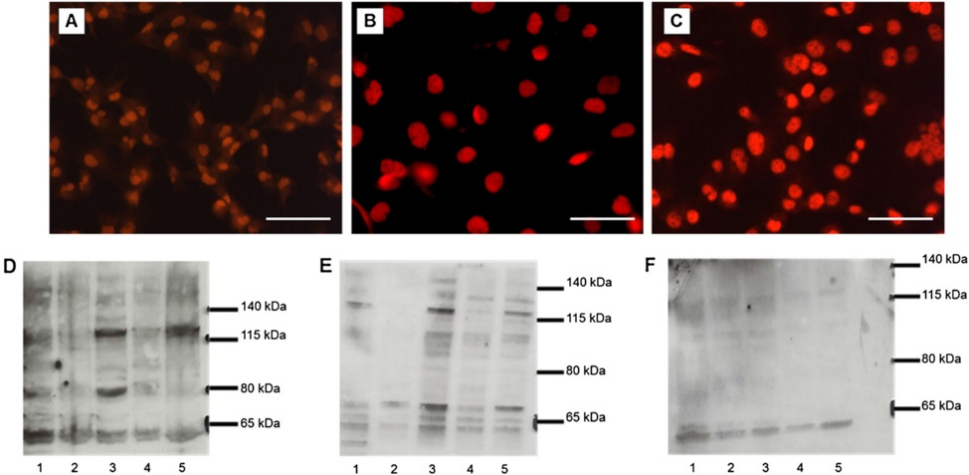
**Detection of HLTF protein by immunofluorescence in 3 thyroid cancer cell lines and Immunodetection of HLTF on Western Blot.** The 3 thyroid cancer cell lines derived from human thyroid cancers: papillary carcinoma B-CPAP **(A)**, follicular carcinoma FTC-133 **(B)** and anaplastic carcinoma 8505C **(C)**. HLTF was detected using the ART2 rabbit antiserum and revealed by a secondary antibody coupled to Alexa Fluor as described in the Methods. The scale bar is 50µm. (D-F) 40µg (E and F) or 60µg **(D)** of cell lysate (total **[D]**, nuclear **[E]** and cytoplasmic **[F]**) were analyzed by 4%-12% gradient PAGE-SDS. After protein transfer to a membrane, immunodetection was performed with the α-HLTF rabbit serum (Sigma, 1/500 **[E and F]** and 1/1000 **[D]**) as described in Methods. Lane 1: TPC-1. Lane 2: B-CPAP. Lane 3: FTC-133. Lane 4: 8505C. Lane 5: HeLa.

We also studied the expression of HLTF protein by immunocytofluorescence in 3 different transformed thyroid cell lines, B-CPAP, FTC-133 and 8505C, derived from human papillary carcinoma, follicular carcinoma and anaplastic carcinoma, respectively. The rabbit anti-serum (ART2) specific for the HLTFMet1 variant was used to determine the localization of HLTF in these cells. HLTF expression was exclusively localized to the nuclear compartment in all cell lines (Figure 7A-C).

We also determined the molecular size of the HLTF protein by Western blotting followed by immunodetection in these cell lines. HeLa cells were used as a positive control because the cancer-associated truncated HLTF protein variants were discovered in these cells [26]. In total cell extracts, a band was detected at approximately 120-kDa in TPC-1, FTC-133, 8505C and HeLa cells (Figure 7D; lanes 1, 3–5). These signals correspond to the HLTF wild-type full-length protein (HLTFMet1). In addition, a second band at 95-kDa, which corresponds to the HLTFMet1ΔB variant, was observed in the TPC1, FTC133 and 8505C thyroid cell lines (Figure 7D; lanes 1, 3–4). Finally, a band at ~80-kDa corresponding to the HLTFMet1ΔA variant was detected in the TPC-1 and FTC-133 thyroid cell lines (Figure 7D; lanes 1 and 3). In the nuclear extracts, the ~120-kDa band was observed in all of the cell lines except for BCPAP (Figure 7E; lanes 1, 3–5). The 95-kDa band was observed in FTC133, 8505C and HeLa cells (Figure 7E; lanes 3–5). We also observed a band at 80-kDa in FTC-133 cells (Figure 7E; lane 3). Finally, a protein was detected at 70-kDa in all cell lines, which may correspond to the HLTFMet123ΔA form, i.e., the translation initiation at the Met 123 codon and the carboxyl-terminal truncation of 274 residues (Figure 7E; lanes 1–5). In the cytoplasmic extracts, two bands were detected at 120- and 95-kDa in all cell lines (Figure 7F, lanes 1–5). In the whole cell extracts, we also observed a 60-kDa band that does not correspond to any known HLTF protein variant (Figure 7D-F; lanes 1–5).

## Discussion

The present study shows for the first time that follicular adenomas present specific nuclear HLTF immunostaining (using the ART2 rabbit anti-serum recognizing the HLTF wild type protein as well as the two HLTF variants, HLTFMet1ΔA and HLTFMet1ΔB), whereas papillary carcinomas exhibit cytoplasmic expression. This observation has been confirmed in a second clinical series of benign and malignant thyroid lesions, allowing us to propose HLTF as a new potential marker of follicular adenomas. This observation could be of major interest for FNAB diagnosis in pathology. In fact, both parameters, LI and MOD, could also be assessed on smeared cells from FNA, but this immunohistochemical quantitative analysis requires computer-assisted microscopy, which is not available in all pathology departments. In this context, we propose further testing of HLTF as a new potential marker of follicular adenomas on FNA using a threshold of 30% HLTF-positive nuclei. In such case, the pathologist could perform a semi-quantitative assessment of HLTF-positive nuclei on smeared cells.

Concerning the difficult differential diagnosis of follicular adenomas from follicular carcinomas, our study showed that HLTF staining was moderate in the cytoplasm and pronounced in the nuclei of follicular adenomas, whereas follicular carcinomas exhibited weak cytoplasmic and nuclear immunostaining. Using quantitative analysis, we demonstrated that HLTF expression decreased significantly when comparing follicular adenomas to follicular carcinomas. However, our results are preliminary, and HLTF expression should be studied in a larger series of follicular adenomas *versus* carcinomas.

Moreover, our *in vivo* study revealed that the expression of one or more truncated HLTF protein variants (HLTFMet1ΔA and HLTFMet1ΔB) was associated with thyroid tumorigenesis. This observation is in agreement with our previous study, which showed that cervical cancers exhibited a significant increase in HLTF expression from normal epithelia to invasive squamous cell carcinoma [27]. We identified the 115-kDa HLTF wild-type protein in cervical intraepithelial neoplasia I-III samples by Western blotting, but only the truncated 83-kDa and 95-kDa proteins were detected in invasive squamous cell carcinoma samples. The 83-kDa and 95-kDa proteins have similar sizes to the HLTF variants Met1ΔA and Met1ΔB, respectively, which were previously characterized in HeLa cells and lack the domains that are involved in DNA repair [26]. In invasive hypopharyngeal squamous-cell carcinomas [26], we also showed that truncated HLTF protein variants increase during tumor progression when comparing carcinomas to normal epithelia or dysplasia and that HLTF overexpression was associated with a worse prognosis.

Considering these data, we propose that these truncated HLTF proteins, having lost important domains that are involved in DNA repair, may contribute to thyrocyte progression in carcinogenesis.

Using immunocytochemistry on several thyroid cancer cell lines, we visualized the HLTF protein exclusively in the nucleus. Nevertheless, wild-type HLTF protein (115-kDa) was present both in the nuclear and cytoplasmic compartments in addition to a truncated form(s) (70-kDa, 83-kDa and/or 95-kDa). Therefore, thyroid cancer cells might utilize various mechanisms to suppress HLTF DNA repair activity: i) mutations affecting the wild type protein activity without excluding it from the nucleus; ii) mutations that shift the end of the reading frame, causing truncated variants; and iii) mutations that alter the nuclear localization signal, causing HLTF exclusion from the nucleus. These alterations could occur by alternative HLTF mRNA splicing, as we have previously observed [26]. In this way, these proteins are unable to perfom DNA repair, which would provide an advantage to cell growth in cancer. Indeed, we have previously shown that truncated HLTF variants are progressively overexpressed during carcinogenesis (head and neck cancer [26] as well as cervical cancer [27]) and replace the wild-type protein. In the immunoblotting experiments presented here, we could not discriminate one type of thyroid cancer from another because all of the cell lines shared the same expression profile of HLTF protein forms. In this regard, van Staveren *et al.*[28] and Saiselet *et al*. [29] have shown by mRNA profiling studies that, regardless of where the cancer originated, thyroid cancer cell lines appear to be the most similar to dedifferentiated thyroid cancer *in vivo* (anaplastic, the most aggressive). Thus, these tumor cell lines are not reliable *in vitro* tools for studying differentiated thyroid tumors because they have progressively acquired characteristics of dedifferentiated cells in culture. However, these cells could be used to identify genes involved in this process of tumor dedifferentiation. Papillary and follicular thyroid cancer cells may have lost their DNA synthesis/replication regulation mechanisms during their *in vitro* cell adaptation, according to the studies cited above [28,29].

Finally, we know that HLTF is implicated in post-replication DNA repair, a mechanism that could be disturbed in thyroid cancer cells [20]. To better understand the role of HLTF in thyroid cancer progression, we would like to study HLTF expression profiles, both at the mRNA level (by RT-PCR) to identify potential mutations and at the protein level.

## Conclusions

This study demonstrates a correlation between HLTF expression level and thyroid neoplastic progression. Nuclear HLTF immunostaining may therefore represent a new marker in thyroid tumors that could be used with FNA in an attempt to better distinguish benign thyroid nodules from malignant tumors. Three truncated HLTF forms lacking the domains that are involved in DNA repair were detected in thyroid carcinomas, strengthening the role of HLTF as a tumor suppressor.

## Abbreviations

FNA: Fine-needle aspiration; HLTF: Helicase-like transcription factor; MOD: Mean optical density; LI: Labelling index; PCNA: Proliferating cell nuclear antigen; HNSCC: Head and neck squamous cell carcinoma; SCC: Squamous cell carcinoma; RT: Room temperature; MI: Mean intensity; Ad: Adenoma; PC: Papillary carcinoma; FC: Follicular carcinoma; AC: Anaplastic carcinoma; CN: Colloid nodule; HT: Hashimoto’s thyroiditis; GD: Grave’s disease; FVPC: Follicular variant of papillary carcinoma.

## Competing interests

The authors declare that they have no competing interests.

## Authors’ contributions

SS and AB conceived and designed the experiments. VA, PP, and LD performed the experiments. VA, PP, LD, CD and MR analyzed the data. GC and NS contributed materials. VA, PP, LD, AB, and SS wrote the paper. All authors read and approved the final manuscript.

## Pre-publication history

The pre-publication history for this paper can be accessed here:

http://www.biomedcentral.com/1471-2407/14/492/prepub

## References

[B1] Jemal A Bray F Center MM Ferlay J Ward E Forman D Global cancer statistics CA Cancer J Clin 2011 61 69 90 21296855 10.3322/caac.20107

[B2] Ding H Descheemaeker K Marynen P Nelles L Carvalho T Carmo-Fonseca M Collen D Belayew A Characterization of a helicase-like transcription factor involved in the expression of the human plasminogen activator inhibitor-1 gene DNA Cell Biol 1996 15 429 442 8672239 10.1089/dna.1996.15.429

[B3] Debauve G Capouillez A Belayew A Saussez S The helicase-like transcription factor and its implication in cancer progression Cell Mol Life Sci 2008 65 591 604 18034322 10.1007/s00018-007-7392-4 PMC11131614

[B4] Blastyak A Hajdu I Unk I Haracska L Role of double-stranded DNA translocase activity of human HLTF in replication of damaged DNA Mol Cell Biol 2010 30 684 693 19948885 10.1128/MCB.00863-09 PMC2812231

[B5] Motegi A Sood R Moinova H Markowitz SD Liu PP Myung K Human SHPRH suppresses genomic instability through proliferating cell nuclear antigen polyubiquitination J Cell Biol 2006 175 703 708 17130289 10.1083/jcb.200606145 PMC2064669

[B6] Unk I Hajdu I Fatyol K Hurwitz J Yoon JH Prakash L Prakash S Haracska L Human HLTF functions as a ubiquitin ligase for proliferating cell nuclear antigen polyubiquitination Proc Natl Acad Sci U S A 2008 105 3768 3773 18316726 10.1073/pnas.0800563105 PMC2268824

[B7] Tian F Sharma S Zou J Lin SY Wang B Rezvani K Wang H Parvin JD Ludwig T Canman CE Zhang D BRCA1 promotes the ubiquitination of PCNA and recruitment of translesion polymerases in response to replication blockade Proc Natl Acad Sci U S A 2013 110 13558 13563 23901102 10.1073/pnas.1306534110 PMC3746927

[B8] Burkovics P Sebesta M Balogh D Haracska L Krejci L Strand invasion by HLTF as a mechanism for template switch in fork rescue 2013 [Epub ahead of print] 10.1093/nar/gkt1040 PMC3919600 24198246

[B9] Helmer RA Foreman O Dertien JS Panchoo M Bhakta SM Chilton BS Role of helicase-like transcription factor (hltf) in the G2/m transition and apoptosis in brain PLoS One 2013 8 e66799 23826137 10.1371/journal.pone.0066799 PMC3691323

[B10] Helmer RA Martínez-Zaguilán R Dertien JS Fulford C Foreman O Peiris V Chilton BS Helicase-like transcription factor (hltf) regulates g2/m transition, wt1/gata4/hif-1a cardiac transcription networks, and collagen biogenesis PLoS One 2013 8 e80461 24278285 10.1371/journal.pone.0080461 PMC3835564

[B11] Bai AH Tong JH To KF Chan MW Man EP Lo KW Lee JF Sung JJ Leung WK Promoter hypermethylation of tumor-related genes in the progression of colorectal neoplasia Int J Cancer 2004 112 846 853 15386372 10.1002/ijc.20485

[B12] Brandes JC van Engeland M Wouters KA Weijenberg MP Herman JG CHFR promoter hypermethylation in colon cancer correlates with the microsatellite instability phenotype Carcinogenesis 2005 26 1152 1156 15760919 10.1093/carcin/bgi058

[B13] Hibi K Nakayama H Kanyama Y Kodera Y Ito K Akiyama S Nakao A Methylation pattern of HLTF gene in digestive tract cancers Int J Cancer 2003 104 433 436 12584739 10.1002/ijc.10985

[B14] Hibi K Nakao A Lymph node metastasis is infrequent in patients with highlymethylated colorectal cancer Anticancer Res 2006 26 55 58 16475679

[B15] Kim YH Petko Z Dzieciatkowski S Lin L Ghiassi M Stain S Chapman WC Washington MK Willis J Markowitz SD Grady WM CpG island methylation of genes accumulates during the adenoma progression step of the multistep pathogenesis of colorectal cancer Genes Chromosomes Cancer 2006 45 781 789 16708352 10.1002/gcc.20341

[B16] Leung WK To KF Man EP Chan MW Bai AH Hui AJ Chan FK Lee JF Sung JJ Detection of epigenetic changes in fecal-DNA as a molecular screening test for colorectal cancer: a feasibility study Clin Chem 2004 50 2179 2182 15502094 10.1373/clinchem.2004.039305

[B17] Leung WK To KF Man EP Chan MW Bai AH Hui AJ Chan FK Sung JJ Quantitative detection of promoter hypermethylation in multiple genes in the serum of patients with colorectal cancer Am J Gastroenterol 2005 100 2274 2279 16181380 10.1111/j.1572-0241.2005.50412.x

[B18] Leung WK To KF Man EP Chan MW Hui AJ Ng SS Lau JY Sung JJ Detection of hypermethylated DNA or cyclooxygenase-2 messenger RNA in fecal samples of patients with colorectal cancer or polyps Am J Gastroenterol 2007 102 1070 1076 17378912 10.1111/j.1572-0241.2007.01108.x

[B19] Moinova HR Chen WD Shen L Smiraglia D Olechnowicz J Ravi L Kasturi L Myeroff L Plass C Parsons R Minna J Willson JK Green SB Issa JP Markowitz HLTF gene silencing in human colon cancer Proc Natl Acad Sci U S A 2009 99 4562 4567 11904375 10.1073/pnas.062459899 PMC123687

[B20] Leung WK Yu J Bai AH Chan MW Chan KK To KF Chan FK Ng EK Chung SC Sung JJ Inactivation of helicase-like transcription factor by promoter hypermethylation in human gastric cancer Mol Carcinog 2003 37 91 97 12766908 10.1002/mc.10124

[B21] Oue N Mitani Y Motoshita J Matsumura S Yoshida K Kuniyasu H Nakayama H Yasui W Accumulation of DNA methylation is associated with tumor stage in gastric cancer Cancer 2006 106 1250 1259 16475210 10.1002/cncr.21754

[B22] Fukuoka T Hibi K Nakao A Aberrant methylation is frequently observed in advanced esophageal squamous cell carcinoma Anticancer Res 2006 26 3333 3335 17094449

[B23] Kang S Kim J Kim HB Shim JW Nam E Kim SH Ahn HJ Choi YP Ding B Song K Cho NH Methylation of p16INK4a is a non-rare event in cervical intraepithelial neoplasia Diagn Mol Pathol 2006 15 74 82 16778587 10.1097/00019606-200606000-00003

[B24] Sandhu S Wu X Nabi Z Rastegar M Kung S Mai S Ding H Loss of HLTF function promotes intestinal carcinogenesis Mol Cancer 2012 11 18 22452792 10.1186/1476-4598-11-18 PMC3337324

[B25] Debauve G Nonclercq D Ribaucour F Wiedig M Gerbaux C Leo O Laurent G Journé F Belayew A Toubeau G Early expression of the Helicase- Like Transcription Factor (HLTF/SMARCA3) in an experimental model of estrogen-induced renal carcinogenesis Mol Cancer 2006 5 23 16762066 10.1186/1476-4598-5-23 PMC1550248

[B26] Capouillez A Debauve G Decaestecker C Filleul O Chevalier D Mortuaire G Coppée F Leroy X Belayew A Saussez S The helicase-like transcription factor is a strong predictor of recurrence in hypopharyngeal but not in laryngeal squamous cell carcinomas Histopathology 2009 55 77 90 19614770 10.1111/j.1365-2559.2009.03330.x

[B27] Capouillez A Noël JC Arafa M Arcolia V Mouallif M Guenin S Delvenne P Belayew A Saussez S Expression of the helicase-like transcription factor and its variants during carcinogenesis of the uterine cervix: implications for tumour progression Histopathology 2011 58 984 988 21585432 10.1111/j.1365-2559.2011.03843.x

[B28] van Staveren WC Solís DW Delys L Duprez L Andry G Franc B Thomas G Libert F Dumont JE Detours V Maenhaut C Human thyroid tumor cell lines derived from different tumor types present a common dedifferentiated phenotype Cancer Res 2007 67 8113 8120 17804723 10.1158/0008-5472.CAN-06-4026

[B29] Saiselet M Floor S Tarabichi M Dom G Hébrant A van Staveren WC Maenhaut C Thyroid cancer cell lines: an overview Front Endocrinol (Lausanne) 2012 3 133 23162534 10.3389/fendo.2012.00133 PMC3499787

